# Aetiology and Potential Animal Exposure in Central Nervous System Infections in Vietnam

**DOI:** 10.1007/s10393-022-01611-w

**Published:** 2022-10-13

**Authors:** Hannah E. Brindle, Behzad Nadjm, Marc Choisy, Rob Christley, Michael Griffiths, Stephen Baker, Juliet E. Bryant, James I. Campbell, Van Vinh Chau Nguyen, Thi Ngoc Diep Nguyen, Ty Thi Hang Vu, Van Hung Nguyen, Bao Long Hoang, Xuan Luat Le, Ha My Pham, Thi Dieu Ngan Ta, Dang Trung Nghia Ho, Thua Nguyen Tran, Thi Han Ny Nguyen, My Phuc Tran, Thi Hong Phuong Pham, Van Tan Le, Dac Thuan Nguyen, Thi Thu Trang Hau, Ngoc Vinh Nguyen, Heiman F. L. Wertheim, Guy E. Thwaites, H. Rogier van Doorn, the VIZIONS Consortium

**Affiliations:** 1 Oxford University Clinical Research Unit, Hanoi, Vietnam; 2 Institute of Infection and Global Health and National Institute, University of Liverpool, Liverpool, UK; 3 Medical Research Council Unit The Gambia at London School of Hygiene and Tropical Medicine, Atlantic Boulevard, Serekunda, The Gambia; 4 Oxford University Clinical Research Unit, Ho Chi Minh City, Vietnam; 5 Centre for Tropical Medicine and Global Health, Nuffield Department of Medicine, University of Oxford, Oxford, UK; 6 Department of Medicine, School of Clinical Medicine, University of Cambridge, Cambridge, UK; 7 The Global Fund to Fight AIDS, Tuberculosis and Malaria, Geneva, Switzerland; 8 Hospital for Tropical Diseases, Ho Chi Minh City, Vietnam; 9 Dak Lak General Hospital, Buon Ma Thuot City, Vietnam; 10 Hanoi Medical University, Hanoi, Vietnam; 11 National Hospital for Tropical Diseases, Hanoi, Vietnam; 12 Wellcome Trust Sanger Institute, Hinxton, UK; 13 Hue Central Hospital, Hue City, Vietnam; 14 Dong Thap General Hospital, Cao Lanh, Vietnam; 15 Khanh Hoa General Hospital, Nha Trang, Vietnam; 16 Research Group 2, AIDS Research Center, National Institute of Infectious Diseases, Tokyo, Japan; 17 Ba Vi District Hospital, Hanoi, Vietnam; 18 RadboudUMC, Nijmegen, The Netherlands

**Keywords:** Zoonosis, Central nervous system infections, Vietnam, Disease of unknown aetiology, Emerging infections

## Abstract

An estimated 73% of emerging infections are zoonotic in origin, with animal contact and encroachment on their habitats increasing the risk of spill-over events. In Vietnam, close exposure to a wide range of animals and animal products can lead to acquisition of zoonotic pathogens, a number of which cause central nervous system (CNS) infections. However, studies show the aetiology of CNS infections remains unknown in around half of cases. We used samples and data from hospitalised patients with CNS infections, enrolled into the Vietnam Initiative on Zoonotic Infections multicentre study, to determine the association between aetiology and animal contact including those in whom the cause was unknown. Among 933 patients, a pathogen or an antibody response to it was identified in 291 (31.2%, 95% CI 28.3–34.3%). The most common pathogens were *Streptococcus suis* (*n* = 91 (9.8%, 8.0–11.9%)) and Japanese encephalitis virus (JEV) (*n* = 72 (7.7%, 6.1–9.7%)). Commonly reported animal contact included keeping, raising or handling (*n* = 364 (39.0%, 35.9–42.2%)) and handling, cooking or consuming raw meat, blood or viscera in the 2 weeks prior to symptom onset (*n* = 371 (39.8%, 36.6–43.0%)), with the latter most commonly from pigs (*n* = 343 (36.9%, 33.8–40.1%). There was no association between an unknown aetiology and exposure to animals in a multivariate logistic regression. Further testing for unknown or undetected pathogens may increase diagnostic yield, however, given the high proportion of zoonotic pathogens and the presence of risk factors, increasing public awareness about zoonoses and preventive measures can be considered.

**Supplementary Information:**

The online version contains supplementary material available at 10.1007/s10393-022-01611-w.

## Introduction

Zoonoses are “diseases that can be transmitted between animals and humans via direct or indirect contacts” (European Centre for Disease Prevention and Control 2022). It is estimated that nearly three-quarters of emerging human pathogens are zoonotic in origin (Woolhouse and Gowtage-Sequeria 2005). Emerging and re-emerging pathogens, which are thought to be driven by factors ranging from change in land use and agriculture to international travel, are more likely to originate from an animal source, particularly in low- and middle-income countries (Woolhouse and Gowtage-Sequeria 2005; Rabaa et al. 2015). Southeast and East Asia are defined as global ‘hotspots’ for the emergence of zoonotic infectious diseases, due to the rapidly growing economies and populations and consequent encroachment on wildlife habitats (Grace et al. 2011). Vietnam is considered to be at risk of spill-over of zoonotic pathogens into humans and has seen a number of emerging pathogens in recent years including avian influenza virus (A/H5N1) (Dinh et al. 2006), and *Streptococcus suis* (Wertheim et al. 2009). Close exposure to livestock and wild animals plays a role in this risk particularly via occupations which involve the handling of raw meat at slaughterhouses or wet markets which often operate with limited biosecurity measures (Carrique-Mas and Bryant 2013). Additionally, food consumption practices, in particular eating raw or undercooked meats and fish and wild animals, are also risk factors for infection with a zoonotic pathogen (Carrique-Mas and Bryant 2013). Following the emergence of SARS-CoV-2, there has been a reinforcement of the regulation of wildlife trade in Vietnam (Borzée et al. 2020).

Central nervous system (CNS) infections in Vietnam are caused by a number of pathogens, many of which are zoonotic (Tan et al. 2014, 2010; Taylor et al. 2012; Trung et al. 2012). Despite the introduction of a vaccination programme in 1997, a common cause of CNS infections in children is Japanese encephalitis virus (JEV) (Tan et al. 2010; Yen et al. 2010). JEV, a flavivirus, is transmitted in an epizootic cycle between infected *Culex* mosquitoes, animal hosts, including pigs and wading birds, and humans (Solomon et al. 2000). The seroprevalence of JEV in pigs in Vietnam ranges from 60% in pigs younger than 6 months of age to 100% in adults (Lindahl et al. 2013; Ruget et al. 2018). The most common cause of bacterial meningitis in adults in Vietnam is *Streptococcus suis*, a Gram-positive bacterium. This infection is associated with eating undercooked pig blood and intestines, or being exposed to bacteria via handling raw pig products (Grace et al. 2011; Wertheim et al. 2009; Nghia et al. 2011). However, in most studies, despite intensive investigations, the aetiology of CNS infections remains unknown in about half of patients—both children and adults (Tan et al. 2014, 2010; Taylor et al. 2012; Trung et al. 2012).

The Vietnam Initiative on Zoonotic Infections (VIZIONS) was developed with the aim of characterizing endemic infections, novel infections and infections of unknown origin through a prospective hospital-based surveillance programme. Patients with either a respiratory tract infection, enteric infection, central nervous system (CNS) infection or jaundice were enrolled as part of the hospital surveillance component (Rabaa et al. 2015). Data from those with CNS infections were used for this study with the aims of (1) determining the aetiology of patients admitted with CNS infections; (2) describing the demographics and geographical distribution of the patients; and (3) understanding whether there is an association between animal contact and CNS infections of known and unknown aetiology.

## Methods

### Patients

**Figure 1:**
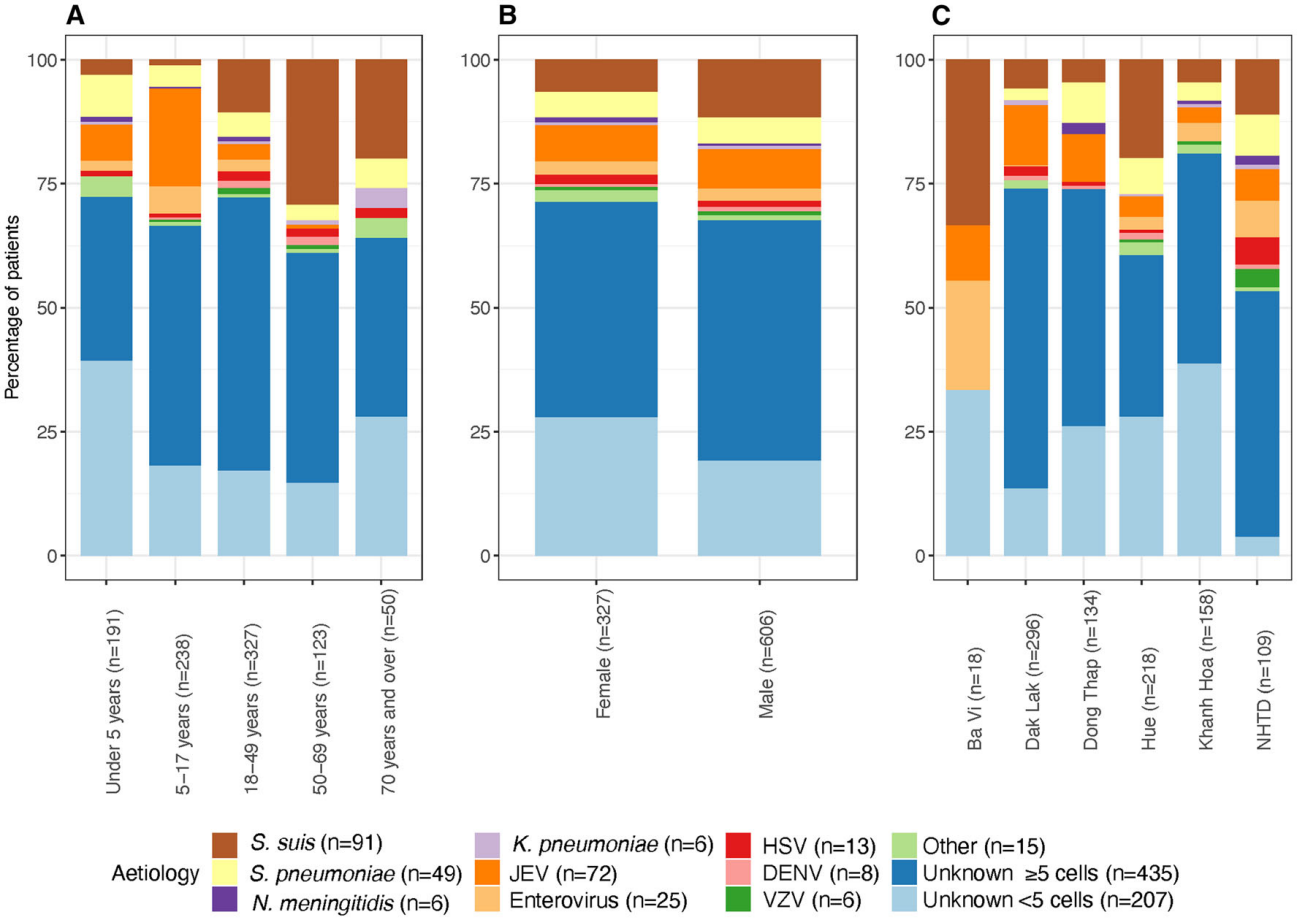
Aetiologies of patients with CNS infection by age category (**A**), gender (**B**) and site of hospital admission (**C**).

Patients aged 1 month and above who were admitted to any of six hospitals across Vietnam including: the National Hospital for Tropical Diseases (NHTD), Ha Noi; Ba Vi District Hospital, Ha Noi (Ba Vi); Hue Central Hospital, Hue (Hue); Dak Lak General Hospital, Buon Me Thuot (Dak Lak); Khanh Hoa General Hospital, Nha Trang (Khanh Hoa); and Dong Thap General Hospital, Cao Lanh (Dong Thap) (Fig. 1) with a suspected CNS infection defined as “fever or a history of fever within the previous three days; presence of one of the following symptoms: headache, neck stiffness, altered consciousness or focal neurological signs; and requiring a diagnostic lumbar puncture as part of clinical care” were enrolled between 1 December 2012 and 21 October 2016. Patients were enrolled from the departments including the intensive care units (adult and paediatrics), infectious diseases, paediatrics, internal medicine and neurology. Patients were excluded if they had a previous hospitalisation within 6 months (adults) and 4 weeks (children) with a CNS infection; or were previously enrolled in the study with a CNS infection (Rabaa et al. 2015).

Informed written consent was obtained from patients (or their parents/legal guardians) included in the study. Diagnostic results from this study were previously described in a paper by Robertson et al. (2020) who looked at the association between contact with pigs and the symptoms and aetiology amongst patients in VIZIONS (Robertson et al. 2020).

### Data Collection

A standard Case Report Form (CRF) detailing demographics, admission to an intensive care unit, self-reported HIV status, drinking water source and animal contact was completed on enrolment (supplementary data). The questions relating to animal exposure included raising, keeping or handling animals; or slaughtering an animal; or handling, cooking or consuming the raw meat, blood or viscera from a list of twenty-nine domestic, livestock and wild animals within the 2 weeks prior to the onset of symptoms. An additional CRF was completed at discharge with the diagnosis and outcome. Global positioning system (GPS) coordinates for the centre of the patients’ commune were obtained using a conversions system via the CliRes Data Management system (https://clires.oucru.org). GPS coordinates were also provided for the location of the hospitals.

### Pathogen Detection

#### Blood and CSF Culture

Clinical specimens including blood and cerebrospinal fluid (CSF) were collected as soon as possible. Initial analysis was undertaken at the hospital of admission including a full blood and CSF cell count, and CSF Gram/Ziehl–Neelsen stain. Blood and CSF culture was performed at the hospital sites including Dak Lak, Dong Thap, Khanh Hoa and Hue with a repeat culture performed at the Oxford University Clinical Research Unit (OUCRU) in Ho Chi Minh City (HCMC) although a blood culture was not obtained for all patients. Discrepancies in culture results between the hospital sites and the putative causal pathogen identified at OUCRU were reviewed to predict the causal pathogen for the purpose of the analysis. In Ba Vi and NHTD, blood and CSF were transported to OUCRU Ha Noi on daily basis where these were cultured.

#### Molecular and Serological Methods

Plasma, blood cells and bacterial isolates stored in 20% glycerol and 1 ml of CSF were transported at − 80 °C on dry ice to the OUCRU in HCMC) or Ha Noi. The Panbio Dengue IgM Capture Enzyme-linked immunosorbent assay (ELISA) (Lu et al. 2019) and InBios JE Detect antibody capture ELISA (Turtle et al. 2019) were performed at the OUCRU Ha Noi on CSF samples from NHTD and Ba Vi District Hospital. The DENV-JEV MACE IgM capture ELISA for the detection of human IgM against dengue viruses (1–4) and JEV (Venture Technologies Sdn Bhd Malaysia) was performed on CSF samples from the other sites at OUCRU HCMC (Trung et al. 2012; Cardosa et al. 2002). Real-time polymerase chain reaction (PCR) was performed for *Neisseria meningitidis, Streptococcus pneumoniae, Streptococcus suis, Haemophilus influenzae* type b, herpes simplex virus (HSV), varicella zoster virus (VZV), and enteroviruses on CSF samples from all hospital sites at OUCRU HCMC (Trung et al. 2012).

The aetiology was determined by the presence of either a positive blood or CSF culture, PCR with a cycle threshold (Ct) value of < 40, or ELISA with an optical density ratio defined as positive according to the manufacturers’ instructions.

### Statistical Analysis

All analyses were performed using R statistical software version 3.6.0 (Core 2020). In the event that repeated specimens were taken, only results from the first were used. Patients in whom no pathogen was found were classified as ‘unknown’ and further categorised into those with a CSF white cell count of < 5 cells/mm^3^ and those with a CSF white cell count of ≥ 5 cells/mm^3^ to account for any potential misdiagnoses of encephalopathy or a CNS infection without a CSF pleocytosis. Age was categorised into five categories: under 5 years; 5–17 years; 18–49 years; 50–69 years and 70 years and over. All the pathogens found in less than six patients were pooled into a category ‘other’. Statistical differences in the demographics of the patients including age category, gender and site of hospital admission by aetiology; and age category and gender by site of hospital admission were determined using Fisher’s exact test and the *p* value simulated with 2000 replicates. Univariate binomial regression was used to calculate the odds ratio (OR) of the presence of each of the aetiologies between age, gender, site of recruitment and type of animal exposure. Univariate binomial regression was also used to calculate the OR between each type of exposure to pigs including keeping, raising or handling; slaughtering or eating, cooking or handling the raw meat, blood or viscera and *S. suis* and JEV.

A mixed-effects multivariate binomial regression model was used to determine the association between any type of animal of exposure and aetiology of the CNS infection. The four most common pathogens, patients with an unknown aetiology and a CSF white cell count of ≥ 5 cells/mm^3^ and a known aetiology; and patients with an unknown aetiology and a CSF white cell count of < 5 cells/mm^3^; age and gender were included as fixed effects. The hospital site was added as a random effect. An outcome of any type of exposure to animals was included as the sample size for the independent variables was too small to perform multivariable logistic regression modelling for exposures including slaughtering an animal or handling, cooking or consuming raw, meat, blood or viscera.

## Results

**Figure 2:**
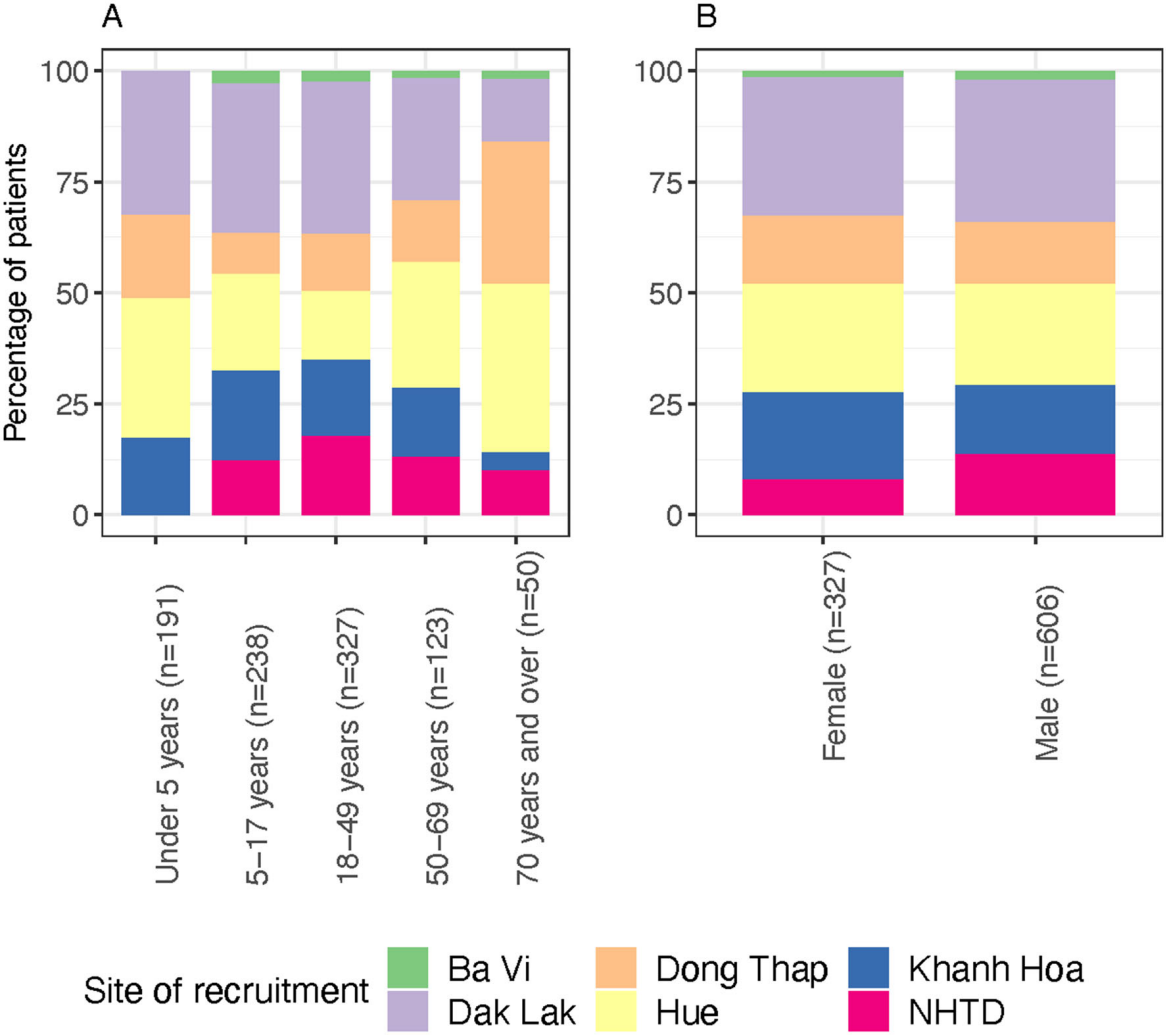
Site of hospital admission by age category (**A**) and gender (**B**).

Of the 969 patients with CNS infections who were enrolled, 36 had more than one pathogen detected and were excluded from the analysis as it was not possible to ascribe cause to a single aetiology. Of the 933 patients included in the analysis, the highest number were recruited from Dak Lak General Hospital (*n* = 296, 31.7%). An aetiology was established in 291/933 (31.2%). Among those in whom a pathogen was detected, 247/291 (87.9%) had CSF white cell count of ≥ 5 cells/mm^3^ and 44/291 (15.1%) had a CSF white cell count of < 5 cells/mm^3^. Among those in whom no pathogen was detected, 435/642 (67.8%) had a CSF white cell count of ≥ 5 cells/mm^3^ and 207/642 (32.2%) had a CSF white cell count of < 5 cells/mm^3^. A total of 12 different bacterial pathogens were found with the most common being *S. suis* (*n* = 91 (9.8%)) and *S. pneumoniae* (*n* = 49 (5.3%)). Of the five viruses tested, the most detected were JEV (*n* = 72 (7.7%)) and enterovirus (*n* = 25 (2.7%)) (Table 1). The median age of the patients was 21 years [inter-quartile range (IQR) = 7–44]. The majority of patients were male (*n* = 606, 65.0%). Infections with *S. suis* were predominantly seen in adult males, with median age of 49 years [IQR = 39–58]. Infections with *S. pneumoniae*, JEV and enterovirus were predominantly seen in paediatric patients, with median age of 12 years [IQR = 2–33 years], 11 years [IQR = 6.8–15] and 13 years [IQR = 8–22], respectively. The median age of those in whom the aetiology was unknown was 23 years [IQR = 10–45] in those with a CSF white cell count of ≥ 5 cells/mm^3^ and 11 years [IQR = 2–36.5] in those with a CSF white cell count of CSF white cell count of < 5 cells/mm^3^. Half of the cases of JEV were recruited from Dak Lak General Hospital (*n* = 36 (50%)) and nearly half of the cases of *S. suis* were recruited from Hue Central Hospital (*n* = 43 (47.3%)). However, although there was statistical evidence of a difference in the aetiology of CNS infection by age of the patient and also, the site of recruitment (*p* = 0.001) there was not by gender of the patient (*p* = 0.062) (Fig. 1 and Table S1, supplementary data). There was evidence of a statistical difference between age groups admitted to the different hospital sites (*p* = 0.001) with a median age ranging from 16 years [IQR = 7–37] in Khanh Hoa to 29.5 years [IQR = 17–42] in the NHTD. No difference was seen between hospital site and gender (*p* = 0.102) (Fig. 2 and Table S2, supplementary data).

**Table 1:** The aetiology of CNS infections in all patients.

Pathogen	Number of patients (*n* = 933) (n (%))
Bacteria	
*Streptococcus suis*	91 (9.8)
*Streptococcus pneumoniae*	49 (5.3)
*Neisseria meningitidis*	6 (0.6)
*Klebsiella pneumoniae*	6 (0.6)
*Haemophilus influenzae* type b	4 (0.4)
*Staphylococcus aureus*	3 (0.3)
*Escherichia coli*	3 (0.3)
*Mycobacterium tuberculosis*	1 (0.1)
*Streptococcus pyogenes*	1 (0.1)
*Pseudomonas aeruginosa*	1 (0.1)
*Streptococcus mitis*	1 (0.1)
*Acinetobacter baumannii*	1 (0.1)
Viruses	
Japanese Encephalitis Virus	72 (7.7)
Enteroviruses	25 (2.7)
Herpes Simplex Virus	13 (1.4)
Dengue viruses	8 (0.9)
Varicella Zoster Virus	6 (0.6)
No pathogen detected	642 (68.8)
Total	291

**Figure 3:**
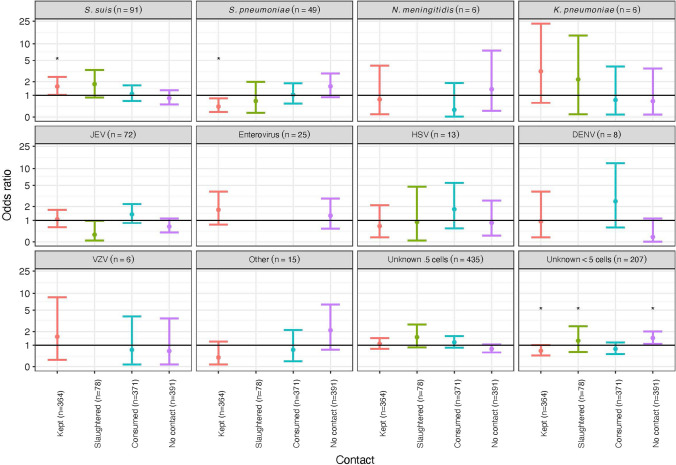
The effect of animal exposure by aetiology as shown by odds ratios with 95% confidence intervals from univariate binomial regression with an outcome of animal contact (kept, slaughtered or consumed).

Three hundred and sixty-four patients (39%) raised an animal, 78 (8.4%) had slaughtered an animal and 371 (39.8%) handled, cooked or consumed raw meat, blood or viscera. In univariate analyses, patients with *S. suis* were more likely to keep, raise or handle an animal compared to those with another pathogen or an unknown aetiology (OR 1.6 (95% CI 1.04–2.48, *p* = 0.033)), and those with *S. pneumoniae* or an unknown aetiology with a CSF white cell count of < 5mm^3^ were less likely to keep, raise or handle an animal (OR 0.44 (0.21–0.83, *p* = 0.017) and OR 0.67 (0.48–0.93, *p* = 0.018), respectively). Those with an unknown aetiology with a CSF white cell count of < 5mm^3^ were less likely to have contact with an animal compared to those with a known pathogen or unknown aetiology with a CSF white cell count of > 5mm^3^ (OR 1.47 (1.08–2), *p* = 0.015)) (Fig. 3 and Table S3, supplementary data). Patients admitted to the hospital in Dak Lak were more likely to raise, keep or handle an animal (OR 1.91 (1.44–2.53), *p* < 0.001), have slaughtered an animal (OR 5.34 (3.28–8.91), *p* < 0.001), and handled, cooked or consumed raw meat, blood or viscera compared to other sites (OR 2.73 (2.06–3.63), *p* < 0.001). Patients admitted to the hospital in Dong Thap were also more likely to have handled, cooked or consumed raw meat, blood or viscera (OR 1.51 (1.04–2.18), *p* = 0.028) whereas those admitted to the hospital in Hue or Khanh Hoa were more likely to have had no contact with an animal (OR 1.82 (1.34–2.47), *p* < 0.001 and OR 2.44 (1.72–3.48), *p* < 0.001, respectively) (Figure S1 and Table S3, supplementary data). Adults aged 50 years and over were more likely to keep, raise or handle animals compared to other age groups (50–69 years: OR 2.43 (1.66–3.59, *p* < 0.001) and 70 years and over: OR 1.92 (1.08–3.42, *p* = 0.026)) with those aged 18–69 years more likely to have slaughtered an animal (18–49 years: OR 3.48 (2.17–5.69, *p* < 0.001) and 50–69 years: 2.5 (1.42–4.27, *p* = 0.001)) and handled, cooked or consumed raw meat, blood or viscera (18–49 years: OR 2.29 (1.74–3.02, *p* < 0.001) and 50–69 years: 1.7 (1.16–2.49, *p* = 0.007). Children aged under 5 years were more likely to have had no contact with animals (OR 2.93, 2.11–4.09, *p* < 0.001) (Figure S2 and Table S3, supplementary data). Females were more likely to have no contact with animals (OR 1.41, 1.08–1.85, *p* = 0.013) with males more likely to keep, raise or handle animals (OR 1.51, 1.14–2.01, *p* = 0.004) but with no difference between the genders in terms of slaughtering animals or handling, cooking or consuming raw meat, blood or viscera (Figure S3 and Table S3, supplementary data).

**Figure 4:**
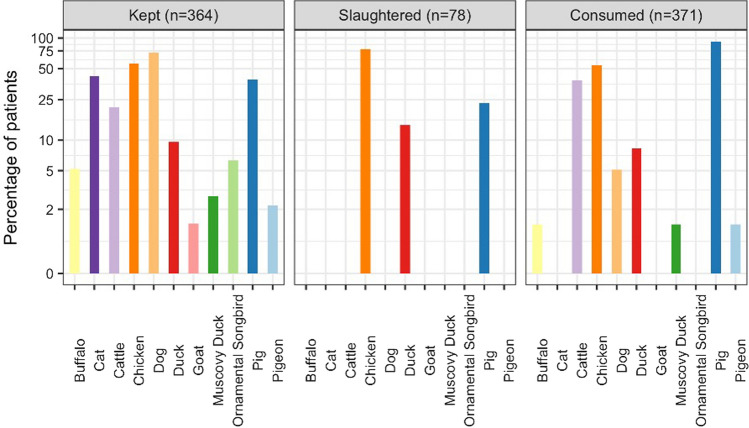
The percentage of patients who had contact with animals by type of animal and type of contact. The figure only includes those animals for which there were five or more patients who had contact. As the patients could have the same contact with more than one type of animal, the percentage is calculated for each type of contact as the number of patients/the sum of the patients who had contact with each animal.

**Figure 5:**
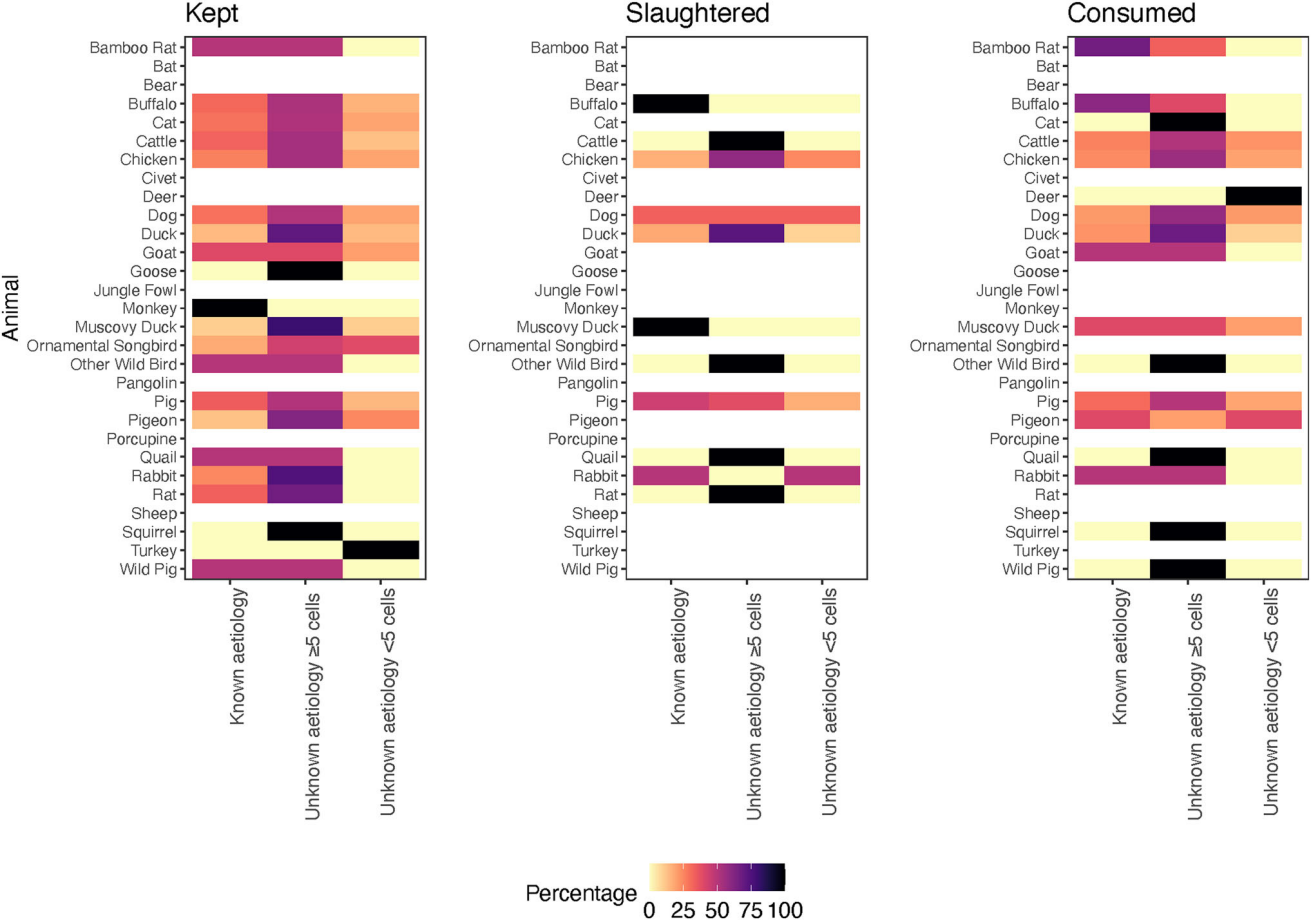
Type of contact by the listed animal and whether the aetiology of the CNS infection was known or unknown. The percentage is according to the row (type of animal).

The most common animals kept, raised or handled were dogs (264/364 patients (72.5%)) and chickens (204/364 patients (56.0%)) both in those with and without a known aetiology. Chickens were the most common animals slaughtered (61/78 patients (78.2%)), and the raw meat, blood or viscera from pigs were the most common product handled, cooked or consumed (*n* = 344/371 (92.7%)) again both in those with and without a known aetiology (Figs. 4 and 5 and Table S4, supplementary data). Wild animals were only slaughtered by those with an unknown aetiology and CSF white cell count of ≥ 5 cells/mm^3^ (other wild bird (0.2%, *n* = 1) and rat (0.2%, *n* = 1)). However, the raw meat, blood or viscera of wild/farmed wild animals were handled, cooked or consumed by those with a known aetiology (bamboo rat (0.7%, *n* = 2) and pigeon (0.7%, *n* = 2)); those with an unknown aetiology and CSF white cell count of ≥ 5 cells/mm^3^ (bamboo rat (0.2%, *n* = 1), other wild bird (0.2%, *n* = 1), pigeon (0.2%, *n* = 1) and squirrel (0.2%, *n* = 1); and those unknown aetiology and CSF white cell count of < 5 cells/mm^3^ (deer (0.5%, *n* = 1) and pigeon (1%, *n* = 2). In the univariate binomial regression, those with *S. suis* were more likely to have slaughtered a pig (OR 4.88, 95% CI 1.66–12.91, *p* = 0.002). However, there was no difference between those with and without *S. suis* who kept or raised pigs; or ate, cooked or handled the raw meat, blood or viscera from pigs (OR 1.19 (95% CI 0.77–1.83, *p* = 0.438) and OR 1.02 (95% CI 0.65–1.59, *p* = 0.918), respectively). No difference was between those with and without JEV and keeping or raising pigs (OR 1.24, 95% CI 0.76–2.01, *p* = 0.383), slaughtering pigs (OR 1.41, 95% CI 0.86–2.28, *p* = 0.167) or eating, cooking or handling the raw meat blood or viscera from pigs (OR 0.70, 95% CI 0.04–3.48, *p* = 0.730).

In the multivariate binomial regression, there was no evidence of a difference between exposure to animals and an aetiology of *S. suis*, *S. pneumoniae*, JEV or enterovirus. Similarly, there was no difference between animal exposure and an unknown aetiology, either with or without a CSF white cell count of ≥ 5 cells/mm^3^. Men were more likely than women to have exposure to animals (OR 1.35, 95% CI 1.00–1.83, *p* = 0.047); however, there was no difference by age (Table 2).

**Table 2:** Multivariate binomial regression model showing the association between the aetiology of the CNS infection and any form of animal exposure including keeping raising or handling an animal, slaughtering an animal or handling, cooking or consuming raw meat, blood or viscera (*n* = 933).

	Odds ratio (95% CI)	*p* value
Intercept	0.34 (0.02–2.88)	
*S. suis* (*n* = 91)	1.23 (0.65–2.74)	0.447
*S. pneumoniae* (*n* = 49)	1.52 (0.68–3.43)	0.312
JEV (*n* = 72)	0.64 (0.29–1.38)	0.250
Enterovirus (*n* = 25)	1.29 (0.48–3.49)	0.614
Unknown aetiology ≥ 5 cells (*n* = 435)	1.03 (0.57–1.89)	0.932
Unknown aetiology < 5 cells (*n* = 207)	1.21 (0.65–2.30)	0.552
Age under 5 years (*n* = 191)	5.41 (0.67–111.14)	0.149
Age 5–17 years (*n* = 238)	2.53 (0.31–51.86)	0.427
Age 18–49 years (*n* = 327)	1.83 (0.23–37.42)	0.604
Age 50–69 years (*n* = 123)	1.34 (0.16–27.69)	0.803
Age 70 years and over (*n* = 50)	1.82 (0.21–38.42)	0.616
Male gender (*n* = 606)	0.75 (0.56–0.99)	0.044

## Discussion

In this multicentre study of CNS infections in Vietnam, an aetiology was not identified in 69% of patients. In a study of adults with a CNS infection of presumed viral aetiology admitted to the Hospital for Tropical Diseases, HCMC, conducted by Tan et al. (2014), the same percentage of patients had an unknown aetiology despite this study also testing for pathogens including cytomegalovirus (CMV), Epstein–Barr Virus (EBV), Nipah virus, influenza A and B virus, Mumps virus, rubella virus, rabies virus and generic flaviviruses which with the exception of JEV and DENV were not included in VIZIONS (Tan et al. 2014). However, the percentage of those with an unknown aetiology in our study was lower than reported by Tan et al. (2010) in the NHTD, Hanoi (73%) (Taylor et al. 2012). Tan et al. (2010) did not, however, test for JEV or DENV but given that they only recruited adults, it is unlikely that the absence of diagnostics for JEV resulted in a much higher proportion of unknown aetiologies. Additionally, although CSF was cultured for mycobacteria if tuberculous meningitis (TBM) was suspected, only 4/352 cases were positive (Taylor et al. 2012).

The percentage of patients with an unknown aetiology in VIZIONS is higher than that found in a multicentre study of CNS infections conducted in provincial hospitals in southern and central Vietnam by Trung et al. (2012) where the aetiology was unknown in 48% of adults and 49% of children (Trung et al. 2012) and a study of children with encephalitis admitted to the Children’s Hospital 1 in HCMC (45%) by Tan et al. (2010). In the study by Trung et al. (2012), more intensive testing for MTB was performed compared to VIZIONS. This included PCR and mycobacterial growth indicator tube (MGIT) culture of the CSF in addition to a smear for acid-fast bacilli (AFB) with 5% (*n* = 34) of adults and 2% (*n* = 11) of children diagnosed with MTB (Nghia et al. 2011). In the study by Tan et al. (2010), PCR was used to detect a wider range of pathogens including CMV, influenza A virus, Me Tri virus, human parechoviruses and generic flaviviruses (Tan et al. 2010). In summary, compared to the four previous studies conducted in Vietnam, we identified fewer patients with a known aetiology compared to two studies, the same number with a known aetiology compared to one study, and more patients with a known aetiology compared to one study.

Pigs were the second most common animals slaughtered and raw pork or pig blood/viscera the most common produced eaten, cooked or handled. The consumption of raw pig blood is common in Vietnam with 35% of respondents in surveys conducted in Hanoi reporting consumption of the dish *Tiet canh* (raw blood pudding) in the previous year (Huong et al. 2014). The risk of meningitis from *S. suis* from infected pigs is common, particularly in Asia (Mai et al. 2008) and another analysis of the VIZIONS data found that over 26% of patients admitted with an enteric, respiratory or CNS infection had had contact with pigs, while eating/handling of raw meat, blood or viscera was the most common form of contact across all hospital sites (Robertson et al. 2020). However, in our study, we only found an association between *S. suis* and slaughtering pigs. In addition to slaughter and consumption, 15% of the patients kept pigs determined as a possible risk factor for JEV in some studies (Liu et al. 2010; Rayamajhi et al. 2007) and Nipah virus (Goh et al. 2000). However, no association was seen in our study.

In addition to contact with livestock and poultry, exposure to wild animals may be associated with novel pathogens, which have the potential to cause CNS infections. Although numbers were very small, 2/4 people with recent history of slaughtering or handling, cooking or consuming of rodents including bamboo rats (*n* = 3), rats (*n* = 1) and squirrels (*n* = 1; one person reported eating both a squirrel and a bamboo rat) had a CNS infection with unknown aetiology and a CSF white cell count of ≥ 5 cells/mm^3^. Rodents are known hosts for a number of zoonotic pathogens with a study in the Mekong delta, Vietnam, detecting antibodies to Tick-borne encephalitis virus (TBEV) and hantavirus in both humans and rodents (Cuong et al. 2015). Rodents are also known hosts of *Leptospira* spp. (Cosson et al. 2014), *Orientia tsutsugamushi* (Wei et al. 2017) and *Rickettsia typhi* (Vallée et al. 2010), which have been determined as causes of CNS infections in Vietnam and neighbouring Lao People’s Democratic Republic (Dittrich et al. 2015; Nadjm et al. 2014).

The final objective of our study was to determine if there was an association between animal contact and a known or unknown aetiology; however, there was no evidence for this, even after adjusting for age, gender and site of recruitment. There was also no association between animal exposure and known zoonotic pathogens such as *S. suis* or JEV, but it is possible that this is attributed to the aggregation of animals and type of exposure. Unfortunately, the small sample size prevented any multivariable analysis by type of exposure. It is also possible that the study was subject to recall bias. While enquiring about the slaughter and consumption of animals within the 2 weeks prior to becoming unwell may minimise recall bias, those with less frequent contact with animals may have been missed, potentially also contributing to the absence of an effect.

Although most common pathogens were tested for in our study, further testing for zoonotic pathogens such as *O. tsutsugamushi* and *Leptospira* spp. and increased diagnostics for MTB could have reduced the proportion of patients in whom no aetiology was identified. This could include quantitative PCR (qPCR) and indirect immunofluorescence assays for *O. tsutsugamushi* and qPCR and microscopic agglutination tests for *Leptospira* spp. as described by Dittrich et al. (2015); and the inclusion of GeneXpert to aid diagnosis of TBM (Bahr et al. 2016).

This study shows that while there was no evidence of a difference between those with and without a CNS infection of unknown aetiology and animal exposure after adjusting for age, gender and site of hospital admission, contact with animals including high-risk contact such as consuming raw meat, blood or viscera is not uncommon and awareness of the risk of infection following these practices should continue to be emphasised. The expansion of testing for pathogens causing CNS infections in the region such as MTB, *O. tsutsugamushi* and *Leptospira* spp. may increase the likelihood of obtaining an aetiology while also enquiring about exposure to rats.

## Supplementary Information

Below is the link to the electronic supplementary material.

Figure S1. The effect of animal exposure by site of recruitment as shown by odds ratios with 95% confidence intervals from univariate binomial regression with an outcome of animal contact (kept, slaughtered or consumed). Figure S2. The effect of animal exposure by age of the patient as shown by odds ratios with 95% confidence intervals from univariate binomial regression with an outcome of animal contact (kept, slaughtered or consumed). Figure S3. The effect of animal exposure by gender of the patient as shown by odds ratios with 95% confidence intervals from univariate binomial regression with an outcome of animal contact (kept, slaughtered or consumed). (DOCX 44 kb)
