# Correction: Aetiology and Potential Animal Exposure in Central Nervous System Infections in Vietnam

**DOI:** 10.1007/s10393-022-01618-3

**Published:** 2022-11-22

**Authors:** Hannah E. Brindle, Behzad Nadjm, Marc Choisy, Rob Christley, Michael Griffiths, Stephen Baker, Juliet E. Bryant, James I. Campbell, Van Vinh Chau Nguyen, Thi Ngoc Diep Nguyen, Ty Thi Hang Vu, Van Hung Nguyen, Bao Long Hoang, Xuan Luat Le, Ha My Pham, Thi Dieu Ngan Ta, Dang Trung Nghia Ho, Thua Nguyen Tran, Thi Han Ny Nguyen, My Phuc Tran, Thi Hong Phuong Pham, Van Tan Le, Dac Thuan Nguyen, Thi Thu Trang Hau, Ngoc Vinh Nguyen, Heiman F. L. Wertheim, Guy E. Thwaites, H. Rogier van Doorn, the VIZIONS Consortium

**Affiliations:** 1 Oxford University Clinical Research Unit, Hanoi, Vietnam; 2 Institute of Infection and Global Health and National Institute, University of Liverpool, Liverpool, UK; 3 Medical Research Council Unit The Gambia at London School of Hygiene and Tropical Medicine, Atlantic Boulevard, Serekunda, The Gambia; 4 Oxford University Clinical Research Unit, Ho Chi Minh City, Vietnam; 5 Centre for Tropical Medicine and Global Health, Nuffield Department of Medicine, University of Oxford, Oxford, UK; 6 Department of Medicine, School of Clinical Medicine, University of Cambridge, Cambridge, UK; 7 The Global Fund to Fight AIDS, Tuberculosis and Malaria, Geneva, Switzerland; 8 Hospital for Tropical Diseases, Ho Chi Minh City, Vietnam; 9 Dak Lak General Hospital, Buon Ma Thuot City, Vietnam; 10 Hanoi Medical University, Hanoi, Vietnam; 11 National Hospital for Tropical Diseases, Hanoi, Vietnam; 12 Wellcome Trust Sanger Institute, Hinxton, UK; 13 Hue Central Hospital, Hue City, Vietnam; 14 Dong Thap General Hospital, Cao Lanh, Vietnam; 15 Khanh Hoa General Hospital, Nha Trang, Vietnam; 16 Research Group 2, AIDS Research Center, National Institute of Infectious Diseases, Tokyo, Japan; 17 Ba Vi District Hospital, Hanoi, Vietnam; 18 RadboudUMC, Nijmegen, The Netherlands

**Correction to: EcoHealth** 10.1007/s10393-022-01611-w

In this article the legend for Figure 1 and 2 was incorrect; the figure should have appeared as shown below. The original article has been corrected.

**Figure 1:**
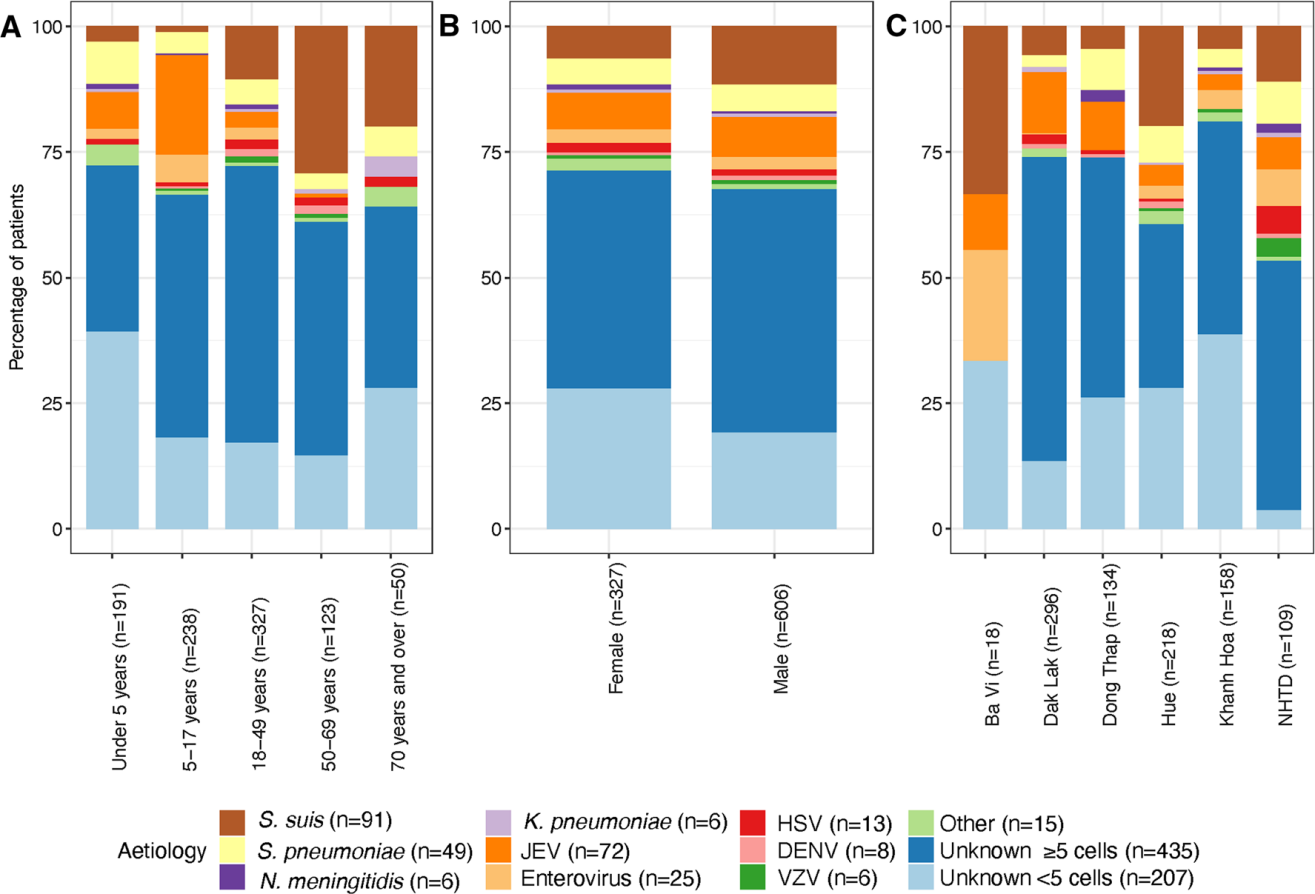
Aetiologies of patients with CNS infection by age category (**A**), gender (**B**) and site of hospital admission (**C**).

**Figure 2:**
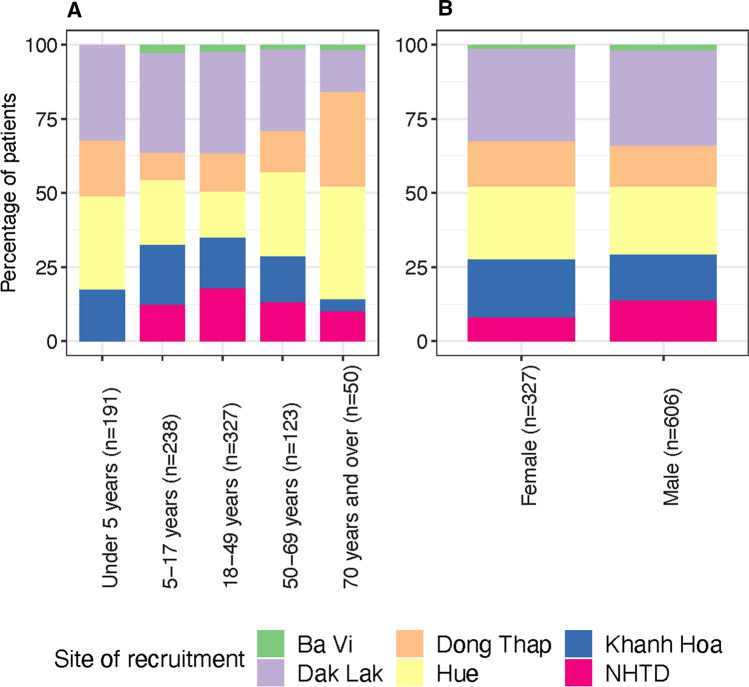
Site of hospital admission by age category (**A**) and gender (**B**).

