# Supplementary material for: Aetiology and Potential Animal Exposure in Central Nervous System Infections in Vietnam
Source: Ecohealth. 2022 Oct 13;19(4):463–74. doi: 10.1007/s10393-022-01611-w (PMC9558024; doi:10.1007/s10393-022-01611-w)
Supplement: Supplementary file 1 — Figure S1. The effect of animal exposure by site of recruitment as shown by odds ratios with 95% confidence intervals from univariate binomial regression with an outcome of animal contact (kept, slaughtered or consumed). Figure S2. The effect of animal exposure by age of the patient as shown by odds ratios with 95% confidence intervals from univariate binomial regression with an outcome of animal contact (kept, slaughtered or consumed). Figure S3. The effect of animal exposure by gender of the patient as shown by odds ratios with 95% confidence intervals from univariate binomial regression with an outcome of animal contact (kept, slaughtered or consumed). (DOCX 44 kb) [file 10393_2022_1611_MOESM1_ESM.docx]

**Supplementary data**

Table S1. Aetiologies of patients with CNS infection by age category, gender and site of hospital admission.

| **Pathogen**  **(n (%))^†^** | ***S. suis* (n=91)** | ***S. pneumoniae* (n=49)** | ***N. meningitidis* (n=6)** | ***K. pneumoniae* (n=6)** | **JEV (n=72)** | **Enterovirus (n=25)** | **HSV (n=13)** | **DENV (n=8)** | **VZV (n=6)** | **Other (n=15)** | **Unknown ≥5 cells**  **(n=435)** | **Unknown <5 cells (n=207)** | **Total (n=933)** | **p value^‡^** |
| --- | --- | --- | --- | --- | --- | --- | --- | --- | --- | --- | --- | --- | --- | --- |
| **Age (years)**  Under 5  5-17  18-49  50-69  70 and over | 6 (3.1)  3 (1.3)  35 (10.7)  36 (29.3)  10 (20.0) | 16 (8.4)  10 (4.2)  16 (4.9)  4 (3.3)  3 (6.0) | 2 (1.0)  1 (0.4)  3 (0.9)  0  0 | 1 (0.5)  0  2 (0.6)  1 (0.8)  2 (4.0) | 14 (7.3)  47 (19.7)  10 (3.1)  1 (0.8)  0 | 4 (2.1)  13 (5.5)  8 (2.4)  0  0 | 2 (1.0)  2 (0.8)  6 (1.8)  2 (1.6)  1 (2.0) | 0  1 (0.4)  5 (1.5)  2 (1.6)  0 | 0  1 (0.4)  4 (1.2)  1 (0.8)  0 | 8 (4.2)  2 (0.8)  2 (0.6)  1 (0.8)  2 (4.0) | 63 (33.0)  115 (48.3)  180 (55.0)  57 (46.3)  18 (36.0) | 75 (39.3)  43 (18.1)  56 (17.1)  18 (14.6)  14 (28.0) | 191  238  327  123  50 | 0.001 |
| **Gender**  Female  Male | 21 (6.4)  70 (11.6) | 17 (5.2)  32 (5.3) | 3 (0.9)  3 (0.5) | 2 (0.6)  4 (0.7) | 24 (7.3)  48 (7.9) | 9 (2.8)  16 (2.6) | 6 (1.8)  7 (1.2) | 2 (0.6)  6 (1.0) | 2 (0.6)  4 (0.7) | 8 (2.4)  7 (1.2) | 142 (43.4)  293 (48.3) | 91 (27.8)  116 (19.1) | 327  606 | 0.068 |
| **Hospital site**  Ba Vi  Dak Lak  Dong Thap  Hue  Khanh Hoa  NHTD | 6 (33.3)  17 (5.7)  6 (4.5)  43 (19.7)  7 (4.4)  12 (11.0) | 0  7 (2.4)  11 (8.2)  16 (7.3)  6 (3.8)  9 (8.3) | 0  0  3 (2.2)  0  1 (0.6)  2 (1.8) | 0  3 (1.0)  0  1 (0.5)  1 (0.6)  1 (0.9) | 2 (11.1)  36 (12.2)  13 (9.7)  9 (4.1)  5 (3.2)  7 (6.4) | 4 (22.2)  1 (0.3)  0  6 (2.8)  6 (3.8)  8 (7.3) | 0  5 (1.7)  1 (0.7)  1 (0.5)  0  6 (5.5) | 0  3 (1.0)  1 (0.7)  3 (1.4)  0  1 (0.9) | 0  0  0  1 (0.5)  1 (0.6)  4 (3.7) | 0  5 (1.7)  0  6 (2.8)  3 (1.9)  1 (0.9) | 0  179 (60.5)  64 (47.8)  71 (32.6)  67 (42.4)  54 (49.5) | 6 (33.3)  40 (13.5)  35 (26.1)  61 (28.0)  61 (38.6)  4 (3.7) | 18  296  134  218  158  109 | 0.001 |

^†^ percentage by row

^‡^ Fisher’s exact test using a simulated p value

Table S2. Site of hospital admission by age category and gender.

| **Pathogen**  **(n (%))^†^** | **Ba Vi (n=18)** | **Dak Lak (n=296)** | **Dong Thap (n=134)** | **Hue (n=218)** | **Khanh Hoa (n=158)** | **NHTD (n=109)** | **Total (n=933)** | **p value^‡^** |
| --- | --- | --- | --- | --- | --- | --- | --- | --- |
| **Age (years)**  Under 5  5-17  18-49  50-69  70 and over | 0  7 (2.9)  8 (2.4)  2 (1.6)  1 (2.0) | 62 (32.5)  80 (33.6)  112 (34.3)  34 (27.6)  7 (14.0) | 36 (18.8)  22 (9.2)  42 (12.8)  17 (13.8)  16 (32.0) | 60 (31.4)  52 (21.8)  51 (15.6)  35 (28.5)  19 (38.0) | 33 (17.3)  48 (20.2)  56 (17.1)  19 (15.4)  2 (4.0) | 0  29 (12.2)  58 (17.7)  16 (13.0)  5 (10.0) | 191  238  327  123  50 | 0.001 |
| **Gender**  Female  Male | 5 (1.5)  13 (2.1) | 102 (31.2)  194 (32.0) | 50 (15.3)  84 (13.9) | 80 (24.5)  138 (22.8) | 64 (19.6)  94 (15.5) | 26 (8.0)  83 (13.7) | 327  606 | 0.094 |

^†^ percentage by row

^‡^ Fisher’s exact test using a simulated p value

Table S3. Type of animal exposure by aetiology of CNS infection.

|  | **Kept, raised or handled an animal (n=364)** | | | **Slaughtered an animal in the two weeks prior to symptom onset (n=78)** | | | **Handled, cooked or consumed raw meat, blood or viscera in the two weeks prior to symptoms onset (n=371)** | | | **No animal contact (n=391)** | | |
| --- | --- | --- | --- | --- | --- | --- | --- | --- | --- | --- | --- | --- |
|  | **Cases (%)** | **OR (95%CI)** ^†^ | **p value**^†^ | **Cases (%)** | **OR (95%CI)** ^†^ | **p value**^†^ | **Cases (%)** | **OR (95%CI)** ^†^ | **p value**^†^ | **Cases (%)** | **OR (95%CI)** ^†^ | **p value**^†^ |
| **Aetiology**  *S. suis* (n=91)  *S. pneumoniae* (n=49)  *N. meningitidis* (n=6)  *K. pneumoniae* (n=6)  JEV (n=72)  Enterovirus (n=25)  HSV (n=13)  DENV (n=8)  VZV (n=6)  Other (n=15)  Unknown ≥5 cells (n=435)  Unknown <5 cells (n=207) | 45 (49.5)  11 (22.4)  2 (33.3)  4 (66.7)  29 (40.3)  13 (52.0)  4 (30.8)  3 (37.5)  3 (50.0)  3 (20.0)  181 (41.6)  66 (31.9) | 1.60 (1.04-2.48)  0.44 (0.21-0.83)  0.78 (0.11-4.02)  3.15 (0.61-22.81)  1.06 (0.64-1.72)  1.72 (0.77-3.86)  0.69 (0.19-2.14)  0.94 (0.19-3.84)  1.57 (0.29-8.51)  0.39 (0.09-1.22)  1.08 (0.80-1.46)  0.70 (0.48-1.01) | 0.033  0.017  0.775  0.187  0.819  0.182  0.542  0.930  0.583  0.142  0.634  0.061 | 12 (13.2)  3 (6.1)  0  1 (16.7)  2 (2.8)  0  1 (7.7)  0  0  0  42 (9.7)  17 (8.2) | 1.79 (0.89-3.34)  0.7 (0.17-1.98)  NA  2.21 (0.11-13.92)  0.3 (0.05-0.97)  NA  0.91 (0.05-4.72)  NA  NA  NA  1.53 (0.88-2.74)  1.28 (0.64-2.53) | 0.084  0.563  NA  0.472  0.093  NA  0.93  NA  NA  NA  0.141  0.479 | 38 (41.8)  20 (40.8)  1 (16.7)  2 (33.3)  33 (45.8)  0  7 (53.9)  5 (62.5)  2 (33.3)  5 (33.3)  187 (43.0)  71 (34.3) | 1.09 (0.70-1.69)  1.04 (0.57-1.86)  0.30 (0.02-1.87)  0.75 (0.10-3.88)  1.38 (0.85-2.24)  NA  1.77 (0.58-5.55)  2.53 (0.62-12.42)  0.75 (0.10-3.88)  0.75 (0.23-2.13)  1.18 (0.87-1.61)  0.80 (0.55-1.16) | 0.699  0.890  0.272  0.744  0.186  NA  0.306  0.205  0.744  0.603  0.275  0.245 | 35 (38.5)  26 (53.1)  3 (50.0)  2 (33.3)  24 (33.3)  12 (48.0)  5 (38.5)  1 (12.5)  2 (33.3)  9 (60.0)  170 (39.1)  102 (49.3) | 0.85 (0.54-1.32)  1.61 (0.90-2.88)  1.39 (0.26-7.54)  0.69 (0.10-3.56)  0.67 (0.40-1.11)  1.29 (0.57-2.87)  0.86 (0.26-2.61)  0.20 (0.01-1.11)  0.69 (0.10-3.56)  2.11 (0.75-6.32)  0.8 (0.62-1.04)  1.47 (1.08-2.00) | 0.483  0.107  0.688  0.671  0.127  0.532  0.800  0.128  0.671  0.161  0.102  0.015 |
| **Site of recruitment**  Ba Vi (n=18)  Dak Lak (n=296)  Dong Thap (n=134)  Hue (n=218)  Khanh Hoa (n=158)  NHTD (n=109) | 18 (100.0)  147 (49.7)  49 (36.6)  52 (23.9)  55 (34.8)  43 (39.4) | NA  1.91 (1.44-2.53)  0.89 (0.60-1.29)  0.40 (0.28-0.57)  0.81 (0.56-1.15)  1.02 (0.68-1.53) | NA  <0.001  0.531  <0.001  0.235  0.921 | 5 (27.8)  53 (17.9)  5 (3.7)  7 (3.2)  4 (2.5)  4 (3.7) | 4.44 (1.39-12.13)  5.34 (3.28-8.92)  0.39 (0.13-0.88)  0.30 (0.12-0.62)  0.25 (0.07-0.60)  0.39 (0.12-0.95) | 0.006  <0.001  0.043  0.003  0.007  0.069 | 8 (44.4)  167 (56.4)  64 (47.8)  70 (32.1)  21 (13.3)  41 (37.6) | 1.21 (0.46-3.10)  2.73 (2.06-3.63)  1.51 (1.04-2.18)  0.65 (0.47-0.89)  0.19 (0.11-0.29)  0.90 (0.59-1.35) | 0.689  <0.001  0.028  0.008  <0.001  0.609 | 0  83 (28.0)  51 (38.1)  116 (53.2)  95 (60.1)  46 (42.2) | NA  0.42 (0.31-0.56)  0.83 (0.57-1.20)  1.82 (1.34-2.47)  2.44 (1.72-3.47)  1.01 (0.67-1.52) | NA  <0.001  0.330  <0.001  <0.001  0.947 |
| **Age (years)**  Under 5 (n=191)  5-17 (n=238)  18-49 (n=327)  50-69 (n=123)  70 and over (n=50) | 30 (15.7)  105 (44.1)  128 (39.1)  71 (57.7)  27 (54.0) | 0.23 (0.15-0.34)  1.34 (0.99-1.81)  1.02 (0.77-1.34)  2.43 (1.66-3.59)  1.92 (1.08-3.42) | <0.001  0.054  0.896  <0.001  0.026 | 0  2 (0.8)  49 (15.0)  20 (16.3)  7 (14.0) | NA  0.07 (0.01-0.22)  3.48 (2.17-5.69)  2.50 (1.42-4.27)  1.85 (0.74-4.03) | <0.001  <0.001  0.001  0.148 | 43 (22.5)  70 (29.4)  173 (52.9)  63 (51.2)  21 (42.0) | 0.36 (0.25-0.52)  0.55 (0.40-0.76)  2.29 (1.74-3.02)  1.70 (1.16-2.49)  1.09 (0.61-1.94) | <0.001  <0.001  <0.001  0.007  0.759 | 120 (62.8)  98 (41.2)  116 (35.5)  37 (30.1)  19 (38.0) | 2.93 (2.11-4.09)  0.96 (0.71-1.29)  0.66 (0.50-0.87)  0.55 (0.36-0.83)  0.84 (0.46-1.50) | <0.001  0.771  0.003  0.004  0.558 |
| **Gender**  Female (n=327)  Male (n=606) | 107 (32.7)  257 (42.4) | 0.66 (0.50-0.87)  1.51 (1.14-2.01) | 0.004  0.004 | 21 (6.4)  57 (9.4) | 0.66 (0.39-1.09)  1.51 (0.91-2.60) | 0.118  0.118 | 123 (37.6)  248 (40.9) | 0.86 (0.66-1.14)  1.16 (0.88-1.53) | 0.301  0.301 | 155 (47.4)  236 (38.9) | 1.41 (1.08-1.85)  0.71 (0.54-0.93) | 0.013  0.013 |

^†^ binomial logistic regression with the presence or absence of each type of animal contact as the dependent variable and the presence or absence of each pathogen as the independent variables. Those with unknown aetiologies were compared to those with any known aetiology.

[Insert Figure S1, here]

[Insert Figure S2, here]

[Insert Figure S3, here]

Table S4. Type of contact by the listed animal and whether the aetiology of the CNS infection was known or unknown. The percentage refers to the number of patients who had contact with each animal/total number of patients with each category of aetiology

|  | **Kept, raised or handled an animal (% (n))** | | | **Slaughtered an animal (% (n))** | | | **Handled, cooked or consumed raw meat, blood or viscera (% (n))** | | |
| --- | --- | --- | --- | --- | --- | --- | --- | --- | --- |
|  | **Known aetiology (n=291)** | **Unknown aetiology ≥5 cells**  **(n=435)** | **Unknown aetiology <5 cells**  **(n=207)** | **Known aetiology**  **(n=291)** | **Unknown aetiology ≥5 cells**  **(435)** | **Unknown aetiology <5 cells**  **(n=207)** | **Known aetiology**  **(n=291)** | **Unknown aetiology ≥5 cells**  **(n=435)** | **Unknown aetiology <5 cells**  **(n=207)** |
| Bamboo Rat  Bat  Bear  Buffalo  Cat  Cattle  Chicken  Civet  Deer  Dog  Duck  Goat  Goose  Jungle Fowl  Monkey  Muscovy Duck  Ornamental Songbird  Other Wild Bird  Pangolin  Pig  Pigeon  Porcupine  Quail  Rabbit  Rat  Sheep  Squirrel  Turkey  Wild Pig | 50.0 (1)  0  0  31.6 (6)  29.4 (45)  32.9 (25)  26.5 (54)  0  0  29.5 (78)  14.3 (5)  40.0 (2)  0  0  100.0 (1)  10.0 (1)  17.4 (4)  50.0 (1)  0  34.3 (49)  12.5 (1)  0  50.0 (1)  25.0 (2)  33.3 (1)  0  0  0  50.0 (1) | 50.0 (1)  0  0  52.6 (10)  51.6 (79)  53.9 (41)  54.4 (111)  0  0  51.5 (136)  71.4 (25)  40.0 (2)  100 (2)  0  0  80.0 (8)  43.5 (10)  50.0 (1)  0  51.0 (73)  62.5 (5)  0  50.0 (1)  75.0 (6)  66.7 (2)  0  100.0 (2)  0  50.0 (1) | 0  0  0  15.8 (3)  19 (29)  13.2 (10)  19.1 (39)  0  0  18.9 (50)  14.3 (5)  20.0 (1)  0  0  0  10.0 (1)  39.1 (9)  0  0  14.7 (21)  25 (2)  0  0  0  0  0  0  100 (1)  0 | 0  0  0  100.0 (1)  0  0  16.4 (10)  0  0  33.3 (1)  18.2 (2)  0  0  0  0  100.0 (1)  0  0  0  44.4 (8)  0  0  0  50.0 (1)  0  0  0  0  0 | 0  0  0  0  0  100.0 (1)  59.0 (36)  0  0  33.3 (1)  72.7 (8)  0  0  0  0  0  0  100.0 (1)  0  38.9 (7)  0  0  100.0 (1)  0  100.0(1)  0  0  0  0 | 0  0  0  0  0  0  24.6 (15)  0  0  33.3 (1)  9.1 (1)  0  0  0  0  0  0  0  0  16.7 (3)  0  0  0  50.0 (1)  0  0  0  0  0 | 66.7 (2)  0  0  60.0 (3)  0  26.1 (37)  24.3 (49)  0  0  21.1 (4)  22.6 (7)  50.0 (1)  0  0  0  40.0 (2)  0  0  0  31.1 (107)  40.0 (2)  0  0  50.0 (1)  0  0  0  0  0 | 33.3 (1)  0  0  40.0 (2)  100.0 (1)  51.4 (73)  56.4 (114)  0  0  57.9 (11)  67.7 (21)  50.0 (1)  0  0  0  40.0 (2)  0  100 (1)  0  50.0 (172)  20.0 (1)  0  100.0 (1)  50.0 (1)  0  0  100.0 (1)  0  100.0 (1) | 0  0  0  0  0  22.5 (32)  19.3 (39)  0  100.0 (1)  21.1 (4)  9.7 (3)  0  0  0  0  20.0 (1)  0  0  0  18.9 (65)  40.0 (2)  0  0  0  0  0  0  0  0 |
